# Effect of Hydroxychloroquine and Azithromycin on QT Interval Prolongation and Other Cardiac Arrhythmias in COVID-19 Confirmed Patients

**DOI:** 10.1155/2021/6683098

**Published:** 2021-02-27

**Authors:** Seyed Parsa Eftekhar, Sohrab Kazemi, Mohammad Barary, Mostafa Javanian, Soheil Ebrahimpour, Naghmeh Ziaei

**Affiliations:** ^1^Student Research Committee, Health Research Center, Babol University of Medical Sciences, Babol, Iran; ^2^Cellular and Molecular Biology Research Center, Health Research Center, Babol University of Medical Sciences, Babol, Iran; ^3^Infectious Diseases and Tropical Medicine Research Center, Health Research Institute, Babol University of Medical Sciences, Babol, Iran; ^4^Department of Cardiology, Babol University of Medical Sciences, Babol, Iran

## Abstract

**Background:**

Hydroxychloroquine with or without azithromycin was one of the common therapies at the beginning of the COVID-19 pandemic. They can prolong QT interval, cause torsade de pointes, and lead to sudden cardiac death. We aimed to assess QT interval prolongation and its risk factors in patients who received hydroxychloroquine with or without azithromycin.

**Methods:**

This study was a retrospective cohort study. One hundred seventy-two confirmed COVID-19 patients were included in this study, hospitalized at Babol University of Medical Sciences hospitals between *March 5, 2020*, and *April 3, 2020*. Patients were divided into two groups: hydroxychloroquine alone and hydroxychloroquine with azithromycin. Electrocardiograms were used for outcome assessment.

**Results:**

83.1% of patients received hydroxychloroquine plus azithromycin vs. 16.9% of patients who received only hydroxychloroquine. The mean age of patients was 59.2 ± 15.4.The mean of posttreatment QTc interval in the monotherapy group was shorter than the mean of posttreatment QTc interval in the combination therapy group, but it had no significant statistical difference (462.5 ± 43.1 milliseconds vs. 464.3 ± 59.1 milliseconds; *p* = 0.488). Generally, 22.1% of patients had a prolonged QTc interval after treatment. Male gender, or baseline QTc ≥ 450 milliseconds, or high-risk Tisdale score increased the likelihood of prolonged QTc interval. Due to QTc prolongation, fourteen patients did not continue therapy after four days.

**Conclusions:**

Hospitalized patients treated by hydroxychloroquine with or without azithromycin had no significant difference in prolongation of QT interval and outcome. The numbers of patients with prolonged QT intervals in this study emphasize careful cardiac monitoring during therapy, especially in high-risk patients.

## 1. Introduction

COVID-19 spread fast and infected millions of people worldwide until now from its beginning in Wuhan, China, and causes high mortality over the world, especially in patients with underlying diseases such as diabetes and congestive heart failure [1, 2]. Since enough clinical trials were unavailable at the early stages of this pandemic, protocols suggested different treatments based on repurposing of medications, clinical trials, *in vitro* studies, and experiences from past coronaviruses like SARS and MERS. However, definite treatment and vaccine need more time and work [3–6]. Remdesivir, hydroxychloroquine sulfate, chloroquine phosphate, and many other drugs showed antiviral efficiency *in vitro*. Even Yao and colleagues suggested hydroxychloroquine is more potent than chloroquine [7, 8]. Shah et al. suggested prophylactic roles for chloroquine and hydroxychloroquine [9]. Nevertheless, clinical trials challenged hydroxychloroquine efficiency, and NIH (National Institutes of Health) denied hydroxychloroquine and chloroquine consumption except in clinical trials [10–12].

Hydroxychloroquine sulfate—which is used for malaria and autoimmune diseases like systemic lupus erythematosus and rheumatoid arthritis—with or without azithromycin was one of the common therapies at the beginning of the pandemic. Studies *in vitro* report hydroxychloroquine could inhibit virus fusion and entry to cell and confronts against the virus [7, 8]. The immunomodulatory effect of hydroxychloroquine is another reason [13, 14]. But a notable concern was raised with this treatment.

Hydroxychloroquine sulfate and macrolides such as azithromycin affect the normal electrophysiologic function of the heart, and concurrent use can cause synergistic effect and may lead to prolonged QT interval, torsades de pointes, and even death [15–18]. Due to the widespread and arbitrary use of these drugs, risk assessment is necessary. However, some studies proceeded on hydroxychloroquine and macrolide effects on QTc interval; still, a question has remained unanswered: does short-term consumption of hydroxychloroquine and azithromycin cause unwanted cardiac effects? We aimed to conduct a retrospective evaluation at Babol University of Medical Sciences hospitals to assess different parameters of electrocardiogram such as QT interval duration and risk factors of QT interval prolongation to find out de novo arrhythmias along with possible electrophysiologic adverse events among hospitalized adult patients with COVID-19. Although similar studies were conducted, we verified their results in Iran and Persian ethnicity. Also, we have a greater sample size than some similar studies, and we looked at other arrhythmias besides the QT interval prolongation.

## 2. Methods and Materials

### 2.1. Data Sources

This study was a retrospective descriptive survey among confirmed COVID-19 adult patients, hospitalized at Babol University of Medical Sciences hospitals. All data and electrocardiograms were extracted from the patients' files. The ethics committee of Babol University of Medical Sciences approved the research.

### 2.2. Patients

We included hospitalized patients between March 5, 2020, and April 3, 2020, and this period was chosen for enough patients with determined discharge status (alive or dead). Inclusion criteria were at least one positive real-time reverse transcriptase polymerase chain reaction test (RT-PCR) of nasopharyngeal samples and age over 18 years old. Exclusion criteria were those aged below eighteen years old, have severe metabolic disease, who refused to receive oral drugs (such as breastfeeding and pregnant mothers), and patients dependent on continuous renal replacement therapy like hemodialysis.

### 2.3. Drug Regimen

All patients received oral hydroxychloroquine sulfate 600 mg daily (200 mg three times daily for ten days), and azithromycin initiated for one hundred forty-one patients (500 mg on day one and 250 mg for the next four days). We divided patients based on the drug regimen into two groups: (1) hydroxychloroquine alone and (2) hydroxychloroquine with azithromycin.

### 2.4. Outcome Assessment

Electrocardiograms were used for the study's outcome assessment, and they were described manually by a cardiologist. Baseline electrocardiograms were recorded at the time of admission. Several electrocardiograms were recorded after treatment, but we used electrocardiograms recorded 3 hours after the second dose of treatment (as posttreatment electrocardiograms) (based on QTc monitoring guidance by Massachusetts General Hospital) [19]. We applied Bazett formula for calculating the QTc interval. The Tisdale Risk Score—which predicts QT interval prolongation risk in hospitalized patients—was used for risk assessment of developing prolonged QT interval.

### 2.5. Statistical Analysis

Proportions were used to represent nominal variables. Means and standard deviations were used to represent continuous variables. *χ*^2^ or Fisher's exact test was used to compare categorical variables, and the odds ratio (ORs) and 95% CI were used to describe them. Continuous variables were compared using the Mann–Whitney *U* test, and *p* value < 0.05 was considered a statistical significance threshold. The logistic regression model was used for evaluating QTc interval prolongation risk (Δ*QTc* ≥ 60 milliseconds or posttreatment QTc interval > 500 milliseconds). SPSS version 20 was used for statistical analysis.

## 3. Results

One hundred forty-three (83.1%) patients received hydroxychloroquine plus azithromycin vs. twenty-nine (16.9%) patients who received only hydroxychloroquine. The mean age of patients was 59.2 ± 15.4. About half of the patients were female. The most common comorbidities were diabetes mellitus, hypertension, and congestive heart failure. More than half of the patients had no history of cardiovascular diseases. The mean of the Tisdale score was 8 ± 2.1, and 12.2% had high-risk scores (score ≥ 11) (Table 1).

**Figure 1:**
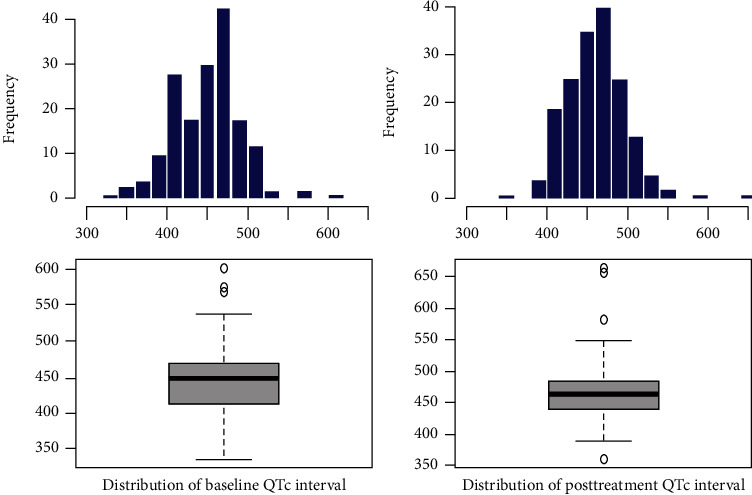
Baseline and posttreatment QTc interval in the monotherapy and combination therapy groups. Hydroxychloroquine alone group mean ± SD values: baseline QTc interval = 459.9 ± 38.3, posttreatment QTc interval = 462.5 ± 43.1, and ΔQTc = 2.6 ± 29.1. Hydroxychloroquine and azithromycin group mean ± SD values: baseline QTc interval = 447.1 ± 44.1, posttreatment QTc interval = 464.3 ± 59.1, and ΔQTc = 17.2 ± 53.4.

The mean of baseline QTc interval—before initiating the treatment—was greater in the monotherapy group than the mean of baseline QTc interval in the combination therapy group, but it was not statistically significant (median ± SD, 459.9 ± 38.3 milliseconds vs. 447.1 ± 44.1 milliseconds; *p* = 0.198). The mean of posttreatment QTc interval in the monotherapy group was shorter than the mean of posttreatment QTc interval in the combination therapy group, but it had no significant statistical difference (median ± SD, 462.5 ± 43.1 milliseconds vs. 464.3 ± 59.1 milliseconds; *p* = 0.488) (Table 2) (Figure 1).

Numbers of posttreatment prolonged QTc intervals (ΔQTc > 60 milliseconds or posttreatment QTc interval ≥ 500 milliseconds) were more significant in the combination therapy group, but it was not statistically significant (thirty-five (24.5%) vs. three (10.3%); *p* = 0.094). Generally, 22.1% of patients had a prolonged QTc interval after treatment (Table 2).

In our sample, nine patients developed an abnormal heart rate after treatment (seven patients with tachycardia and two patients with bradycardia). Other events such as atrial fibrillation (three patients), premature atrial contraction (one patient), ST-segment elevation (four patients), and bundle branch blocks (one patient with right bundle branch block and three patients with left bundle branch block) occurred with low incidence, and there was no significant statistical difference between two groups (Table 3).

Two patients developed ventricular tachycardia. Both of them were in a combination therapy group. Two cases of torsade de pointes occurred (one in the monotherapy group and one in the combination therapy group) (Table 3).

Male gender (27 of 87 patients (31.1%) vs. 11 of 85 patients (12.9%); *p* = 0.005), or baseline QTc ≥ 450 milliseconds (23 of 79 patients (29.1%) vs. 15 of 93 patients (16.1%); *p* = 0.43), or high-risk Tisdale score (7 of 17 patients (41.2%) vs. 31 of 155 patients (20%); *p* < 0.001) increased likelihood of prolonged QTc interval (QTc ≥ 500 millisecond or ΔQTc > 60 millisecond). Also, in the adjusted model of the three above risk factors, male gender and high-risk Tisdale score remained independently associated with the occurrence of prolonged QTc interval. Underlying diseases and QTc prolonging agents' consumption did not correlate with prolonged QTc interval (Table 4).

One hundred forty-one patients were discharged and thirty-one patients died; it shows no significant statistical difference between the outcomes of the monotherapy group and combination therapy group (4 deaths and 25 discharged in monotherapy group vs. 27 deaths and 116 discharged in combination therapy group; *p* = 0.516).

Due to QTc prolongation, fourteen patients did not continue hydroxychloroquine and azithromycin after four days. Fifteen patients developed transient nausea and dizziness that may be due to the side effects of hydroxychloroquine.

## 4. Discussion

We found that the duration of the QTc interval prolongation was greater in the combination therapy group than in the monotherapy group. However, it was not statistically significant, and it seems that concerns of arrhythmogenic events of azithromycin should not be determinative on choosing the drug regimen. Male gender, baseline QTc interval ≥ 450 milliseconds, and high-risk Tisdale score increased QTc interval prolongation risks. Underlying diseases such as diabetes mellitus, congestive heart failure, and hypertension did not affect QT interval prolongation. Few patients developed conditions such as tachycardia, bradycardia, atrial fibrillation, ST-segment elevation, and bundle branch blocks, but the study suggests that their incidence was not impressive, and two groups had no significant statistical difference.

We did not find an influential role for torsade de pointes and ventricular tachycardia as a risk factor on patients' outcomes (in the monotherapy group and combination therapy group).

Hydroxychloroquine and azithromycin combination was common at the beginning of the pandemic: studies *in vitro* report hydroxychloroquine could inhibit virus fusion and entry to cell and confronts against the virus [7, 8]. But a notable concern was raised with this treatment: is the short-term administration of these drugs increasing the risk of QT interval prolongation and torsade the pointes?

Many studies introduce antimalarial drugs as QT interval prolonging agents (such as quinidine and halofantrine) [20, 21]. The function and structure of hydroxychloroquine are similar to quinidine (a class IA antiarrhythmic agent). Hydroxychloroquine can block the human ether-à-go-go-related gene (hERG) potassium channel, prolong QT interval, and increase the risk of torsade de pointes, which could lead to sudden cardiac death [22]. Hydroxychloroquine chronic consumption may lead to QT interval prolongation and torsade de pointes [23, 24].

Azithromycin—a widely prescribed antibiotic—caused QT prolongation in many cases [25, 26]. In 2012, Ray et al. reported azithromycin (within five days) increased sudden cardiac death (its effect was small but statistically significant); after stopping the treatment, this effect did not persist [15]. Increasing the intracellular sodium ion and dysregulation of intracellular calcium may be the probable mechanism [27].

Mercuro et al. found 23% of patients who received hydroxychloroquine with or without azithromycin developed QT interval prolongation, and one patient developed torsade de pointes [28]. Saleh et al. reported—among 201 patients treated with hydroxychloroquine and azithromycin—that the duration of QT prolongation was greater in the combination therapy group than in the hydroxychloroquine alone group, and none of the patients developed torsade de pointes [29]. Rosenberg et al. showed that the most common adverse event of hydroxychloroquine alone or with azithromycin was abnormal electrocardiogram findings, and the combination therapy group and monotherapy group had no significant difference [11]. Our results are consistent with the above findings in some aspects, such as we found 22.1% of patients developed prolonged QT interval and it was the most common adverse event, even though we did not find a significant difference in the occurring QTc interval prolongation between the combination therapy group and the monotherapy group.

We found that the male gender is a risk factor for QT prolongation; however, a previous study suggested that the female gender is a risk factor for adverse drug reactions [30]. The novel coronavirus's effect may cause this variation at heart that we did not evaluate by a control group.

QTc interval prolongation may lead to torsade de pointes, but it is not a definitive predictive factor for torsade de pointes occurrence. Among QT prolonging agents, antiarrhythmic drugs have a greater role in causing torsade de pointes (1-5%) than noncardiac drugs (0.001%) [31]. In our study, two patients developed torsade de pointes, and the incidence of torsade de pointes between the combination therapy group and monotherapy group did not differ significantly.

Our findings suggest that the prescription of hydroxychloroquine and azithromycin needs careful cardiac monitoring, especially in males, patients with high-risk Tisdale scores, and patients with baseline QTc interval ≥ 450 milliseconds. These results are consistent with the American College of Cardiology's recommendation about baseline risk assessment and careful QTc interval monitoring; also, NIH states similar concerns by limiting the high-dose prescription of these drugs to clinical trials [12, 32].

## 5. Limitations

It seems hydroxychloroquine and azithromycin play a role in the QT interval prolongation, but our main limitation was the absence of the control group for evaluating the electrophysiologic effects of COVID-19. The numbers of patients in this study were fewer than the numbers of the patients in the initial phases of the COVID-19 pandemic treated with hydroxychloroquine with or without azithromycin. We evaluate hospitalized patients, and we did not follow them after discharge to find out long-term adverse drug reactions.

## 6. Conclusion

Hospitalized patients treated by hydroxychloroquine with or without azithromycin had no significant difference in the prolongation of QT interval and outcome. However, numbers of patients with prolonged QT intervals in this study emphasize careful cardiac monitoring during therapy, especially in high-risk patients. Further studies and clinical trials are needed to evaluate these drugs' effectiveness on the outcome and possible adverse drug reactions.

## Figures and Tables

**Table 1:** COVID-19 patients' characteristics at the time of administration.

Characteristic	Total (*n* = 172) (%)	Hydroxychloroquine (*n* = 29) (16.9%)	Hydroxychloroquine and azithromycin (*n* = 143) (83.1%)	*p* value
Age, mean ± SD (year)	59.2 ± 15.4	59.1 ± 17.9	59.8 ± 14.8	0.577
Female	85 (49.4)	16 (55.1)	69 (48.2)	0.497
Intensive care at time of testing	17 (9.8)	3 (10.3)	14 (9.7)	0.927
Tisdale score at treatment initiation, mean ± SD	8 ± 2.1	5.8 ± 2.3	8.4 ± 1.8	<0.001
Acute heart failure	28 (16.2)	6 (20.6)	22 (15.3)	0.480
Maximum temperature at the time of administration, mean ± SD (°C)	37.1 ± 0.5	37.1 ± 0.4	37.2 ± 0.5	0.192
Baseline laboratory values, mean ± SD				
Serum potassium, mean ± SD (mEq/L)	4.2 ± 0.3	4.3 ± 0.3	4.1 ± 0.3	0.062
Serum creatinine, mean ± SD (mg/dL)	1.2 ± 1.1	1.1 ± 0.3	1.2 ± 1.2	0.810
White blood cell count, mean ± SD (cells/*μ*L)	7140.1 ± 3501.4	7848.6 ± 4880.4	6995.2 ± 3128.1	0.627
Absolute lymphocyte count, mean ± SD (count/*μ*L)	22.78 ± 9.6	23.1 ± 12.3	22.71 ± 9.1	0.325
C-reactive protein, mean ± SD (mg/dL)	8.8 ± 5.9	9.6 ± 7.4	8.6 ± 5.6	0.708
Creatine phosphokinase, mean ± SD (IU/L)	232.4 ± 395.5	146.4 ± 55.9	272.5 ± 473.7	0.940
Lactate dehydrogenase, mean ± SD (IU/L)	642.9 ± 358.7	507.2 ± 219.3	679.1 ± 380.3	0.003
Preexisting conditions				
Hypertension	53 (30.8)	10 (34.4)	43 (30.1)	0.639
Congestive heart failure	48 (27.9)	4 (13.7)	44 (30.7)	0.063
Coronary artery disease	11 (6.3)	0 (0)	11 (7.6)	0.123
Atrial fibrillation	2 (1.2)	1 (3.4)	1 (0.7)	0.208
Diabetes mellitus	60 (34.8)	7 (24.1)	53 (37.1)	0.183
Chronic obstructive pulmonary disease	4 (2.3)	0 (0)	4 (2.7)	0.362
Malignancy	7 (4.1)	2 (6.8)	5 (3.4)	0.398
Chronic kidney disease	10 (5.8)	1 (3.4)	9 (6.2)	0.550
Medications				
Loop diuretics	41 (23.8)	8 (27.5)	33 (23.1)	0.603

Abbreviations: SD: standard deviation.

**Table 2:** Baseline and posttreatment QTc interval.

Characteristic	Total (*n* = 172) (%)	Hydroxychloroquine (*n* = 29) (%)	Hydroxychloroquine and azithromycin (*n* = 143) (%)	*p* value
Baseline QTc, mean ± SD (ms)	449.1 ± 43.3	459.9 ± 38.3	447.1 ± 44.1	0.198
Posttreatment QTc, mean ± SD (ms)	464.1 ± 56.6	462.5 ± 43.1	464.3 ± 59.1	0.488
*Δ*QTc, mean ± SD (ms)	15 ± 50.3	2.6 ± 29.1	17.2 ± 53.4	0.463
Prolonged QTc	38 (22.1)	3 (10.3)	35 (24.5)	0.094

Abbreviations: SD: standard deviation; QTc: corrected QT interval; *Δ*QTc: change in corrected QT interval; ms: millisecond.

**Table 3:** Posttreatment electrocardiographic findings of COVID-19 patients.

Findings	Total (*n* = 172) (%)	Hydroxychloroquine (*n* = 29) (%)	Hydroxychloroquine and azithromycin (*n* = 143) (%)	*p* value
Heart rate				
Normal	163 (94.7)	28 (96.5)	135 (94.4)	0.636
Tachycardia	7 (4.1)	0 (0)	7 (4.9)	0.224
Bradycardia	2 (1.2)	1 (3.5)	1 (0.7)	0.208
Supraventricular				
Atrial fibrillation	3 (1.7)	1 (3.4)	2 (1.4)	0.208
PAC	1 (0.6)	0 (0)	1 (0.7)	0.652
Ventricular				
Ventricular tachycardia	2 (1.2)	0 (0)	2 (1.4)	0.522
TdP	2 (1.2)	1 (3.4)	1 (0.7)	0.208
ST segment elevation	4 (2.3)	0 (0)	4 (28)	0.362
Bundle branch block				
LBBB	1 (0.6)	0 (0)	1 (0.7)	0.652
RBBB	3 (1.7)	1 (3.4)	2 (1.4)	0.442

Abbreviations: PAC: premature atrial contraction; TdP: torsade de pointes; RBBB: right bundle branch block; LBBB: left bundle branch block.

**Table 4:** Prolonged QTc interval (corrected QT interval) risk assessment.

		Prolonged QTc interval			
Characteristic	Patient numbers (%)	Odds ratio (95% CI)	*p* value	Adjusted odds ratio (95% CI)	*p* value
Male	85 (49.4)	3.027 (1.389–6.600)	0.005	4.113 (1.772–9.545)	0.001
Baseline QTc ≥ 450 ms	79 (45.9)	2.136 (1.024–4.456)	0.043	1.681 (0.749–3.774)	0.208
Congestive heart failure	48 (27.9)	1.068 (0.481–2.369)	0.871	NT	NT
Hypertension	53 (30.8)	0.527 (0.224–1.244)	0.144	NT	NT
Coronary artery disease	11 (6.3)	2.134 (0.590–7.719)	0.248	NT	NT
Diabetes mellitus	60 (34.8)	0.827 (0.383–1.786)	0.628	NT	NT
Chronic obstructive pulmonary disease	4 (2.3)	3.667 (0.499–26.639)	0.202	NT	NT
Chronic kidney disease	10 (5.8)	1.555 (0.382–6.327)	0.537	NT	NT
QT prolonging agents					
1 QT prolonging agent	29 (16.9)	1		NT	NT
≥2 QT prolonging agent	143 (83.1)	2.809 (0.801–9.847)	0.107	NT	NT
Tisdale score					
Low risk (<7)	43 (25)	1		1	
Moderate risk (7–11)	111 (64.5)	1.793 (0.679–4.733)	0.239	2.196 (0.764–6.311)	0.144
High risk (≥11)	17 (9.9)	4.317 (1.182–15.760)	0.027	6.369 (1.467–27.600)	0.013

Abbreviation: ms: milliseconds; QTc: corrected QT interval; CI: confidence interval; NT: not tested.

## Data Availability

All data available from corresponding author upon request.
